# Role of the Glycine Transporter GlyT2 in the Neuronal Differentiation of PC12 Cells

**DOI:** 10.3390/ijms27073026

**Published:** 2026-03-26

**Authors:** Jorge Sarmiento-Jiménez, Beatriz Morales-González, Enrique Núñez, Elena Martínez-Blanco, Francisco Zafra, Francisco Javier Díez-Guerra, Beatriz López-Corcuera

**Affiliations:** 1 Departamento de Biología Molecular, Instituto de Biología Molecular (IUBM), Centro de Biología Molecular “Severo Ochoa”, Consejo Superior de Investigaciones Científicas-Universidad Autónoma de Madrid, 28049 Madrid, Spain; jsarmiento@cbm.csic.es (J.S.-J.); b.moralesgonzalez@student.vu.nl (B.M.-G.); enrinubal@gmail.com (E.N.); elena.martinez@cbm.csic.es (E.M.-B.); fzafra@cbm.csic.es (F.Z.); fjdiez@cbm.csic.es (F.J.D.-G.); 2 IdiPAZ-Hospital Universitario La Paz, 28046 Madrid, Spain

**Keywords:** glycine transport, GlyT2, hyperekplexia, development, neuron differentiation, PC12 cells, GAP-43

## Abstract

Hyperekplexia is a neurologic disorder of marked perinatal significance. Affected neonates display generalized hypertonia and exaggerated startle reflex in response to innocuous stimuli, potentially leading to life-threatening apneic episodes. Although symptom severity typically diminishes during the first year of life, affected individuals often continue to exhibit disabling motor dysfunction and frequent unprotected falls throughout adulthood. Currently, no targeted therapeutic interventions are available. The pathophysiology involves partial or complete disruption of inhibitory glycinergic neurotransmission. Mutations in the gene encoding the neuronal glycine transporter GlyT2 (*SLC6A5*) represent the second-most frequent genetic etiology of human hyperekplexia. To investigate the mechanistic basis for the heightened severity of symptoms during the perinatal period, we examined the role of GlyT2 in neuronal differentiation using the PC12 cell model. Pharmacological induction of differentiation demonstrated that clones stably expressing GFP-GlyT2 exhibit increased expression of neuronal differentiation markers and enhanced neurite outgrowth—both in number and length—relative to parental PC12 cells. These clones also displayed elevated cytosolic calcium levels, which were attenuated by calmodulin overexpression, subsequently downregulating differentiation marker expression. We hereby proved that GlyT2 is clearly implicated in growth cone progression and differentiation of PC12 cells into neurons by increasing internal calcium and binding to growth cone proteins. Finally, our results were validated in primary neurons.

## 1. Introduction

Glycine, the main inhibitory neurotransmitter in the brain stem and spinal cord, regulates muscle tone, motor rhythms, reflex responses, and sensory information, including visual, acoustic and nociceptive transmission. In glycinergic pathways, synaptic glycine released by glycinergic interneurons activates glycine receptors (GlyR), which are ligand-gated chloride channels that mediate synaptic inhibition [1]. The neuronal glycine transporter GlyT2 removes glycine from the synaptic cleft through active Na^+^, Cl^−^ and glycine cotransport, assisting its glial counterpart GlyT1 in the termination of the glycinergic transmission [2]. GlyT2 transport fulfills an additional crucial role supplying enough glycine to the presynapse to facilitate the refilling of synaptic vesicles. Disrupting GlyT2 function by gene deletion in mice impedes glycinergic neurotransmission and reproduces the symptoms of a human disease called hyperekplexia (HPX) [3].

HPX or startle disease (OMIM149400, 614619, 614618, 618011) is a group of rare neurological syndromes characterized by an overactive startle reflex controlled by glycinergic neurons. Newborns with HPX exhibit intense spasms and stiffness triggered by unexpected trivial stimuli, posing risks to swallowing and breathing. Infants may suffer brain damage or even sudden death due to apnea episodes. Symptoms often improve during infancy, indicating a perinatal window for the disease. However, the exaggerated startle response persists into adulthood, resulting in ongoing motor difficulties and recurrent falls [4]. Presently, no specific treatment exists for HPX, and therapy involves enhancing GABAergic neurotransmission using benzodiazepines (clonazepam). This treatment is not exempted of side effects such as sedation [5].

About 25% of HPX cases reported in the literature affect the human GlyT2 gene (*SLC6A5*), with the majority (60%) affecting the α1 subunit of the GlyR gene *GLRA1* [6]. Around 50 mutations in the *SLC6A5* gene have been identified, some of which have been studied and their pathogenic mechanisms analyzed [7,8,9]. The cognitive function of HPX patients is usually normal. However, recent studies have shown that learning difficulties, delayed speech acquisition and developmental delay are present in 40–80% of patients, supporting a role of glycinergic transmission in neurocognitive development [6,10].

Glycinergic activity has been shown to have a role in proliferation, cell specification and circuit formation during the CNS development [11]. In zebrafish, glycine signaling was identified as regulator of neurogenesis in the spinal cord [12], and paracrine release of glycine was found to be necessary and sufficient to drive correct interneuron differentiation [13] through calcium transients in neural progenitors [14]. In rodents, evidence indicates that glycine release at embryonic day 12.5 (E12.5) modulates the propagation of the spontaneous activity in the isolated embryonic spinal cord [15].

GlyRs including the embryonic α2 subunits are involved in the regulation of interneuron differentiation during spinal cord development both in zebrafish [16] and in mice [17]. Moreover, the expression of GlyT2 precedes the establishment of glycinergic activity in multiple central auditory regions in the rat, suggesting GlyT2 function influences the development of inhibitory networks [18]. In rat primary spinal cord neurons and in zebrafish embryos, GlyT2 expression is downregulated by Hedgehog signaling, a pathway that plays a crucial role in brain development [19]. Furthermore, HPX patients with GlyT2 gene mutations were significantly more likely to experience recurrent infantile apneas and developmental delay than those containing *GLRA1* mutations [5,6]. This, together with the heightened severity of HPX symptoms during the perinatal period, reinforces the suggestion of a role for GlyT2 in the development of glycinergic neurotransmission. Here, we study the role of GlyT2 in neuronal differentiation using PC12 cells as experimental system.

The PC12 cell line, derived from rat adrenal pheochromocytoma, have provided a robust system for investigating the molecular mechanisms underlying the conversion of progenitor-like cells into functionally differentiated neurons [20]. Upon nerve growth factor (NGF) treatment, PC12 cells arrest proliferation, extend neurite-like processes, and adopt morphological and gene expression changes characteristic of sympathetic-like neurons [21]. This NGF-induced differentiation paradigm is widely used to study neurogenesis, neuronal survival, and plasticity [22].

During NGF-induced differentiation, the ERK–CREB signaling axis serves as a central node translating neurotrophic stimulation into long-lasting transcriptional programs that establish the neuronal phenotype [23]. In PC12 cells, sustained ERK activation facilitates stable neuronal differentiation, whereas transient ERK activation drives proliferative responses [24]. In parallel, NGF-induced differentiation drives cytoskeletal remodeling that supports neurite extension [25]. Differentiation also enhances the expression and trafficking of synaptic vesicle proteins, including synaptophysin and synaptotagmin, reflecting functional maturation of the neuronal phenotype [26]. Considering PC12 cells are a well-established model of robust NGF responsiveness, ease of culture, and amenability to genetic manipulation [27], we used this system to investigate the role of GlyT2 in neurite outgrowth and neuronal maturation. Our results demonstrate that GlyT2 is directly involved in growth cone progression and facilitates neuronal differentiation by increasing internal calcium and interacting with growth cone proteins. We validated this GlyT2 role in primary neurons, which enhances the translational relevance of our findings.

## 2. Results

### 2.1. GAP-43 as a Marker of Growth Cones in PC12 Cells

**Figure 1:**
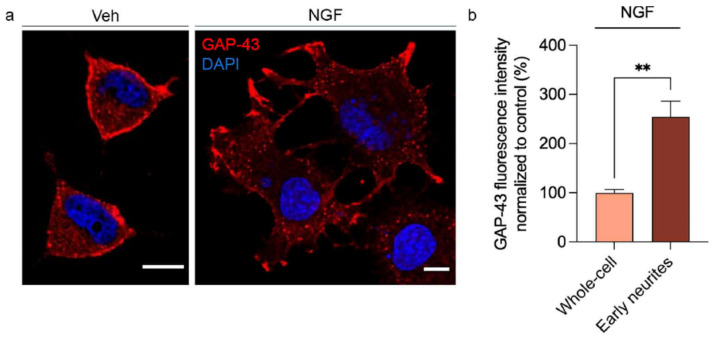
GAP-43 redistributes to early neurites during NGF-induced differentiation of PC12 cells. (**a**) Undifferentiated PC12 cells (Vehicle, Veh) and cells treated with NGF (100 ng/mL, 24 h) were analyzed by immunofluorescence to visualize the subcellular distribution of GAP-43. Upon NGF stimulation, GAP-43 relocates from the plasma membrane to emerging neuritic extensions. Scale bars: 10 µm. (**b**) Quantification of GAP-43 fluorescence intensity in whole-cell and early neurite regions. Values are normalized to the mean intensity measured in whole-cell areas and expressed per unit area (set to 100%). ** *p* < 0.01 indicates significant difference from control in a Student’s *t*-test analysis (38 cells counted in each of *n =* 6 independent cell culture preparations).

PC12 cells provide a well-established model to study neuronal differentiation, as they undergo a reproducible transition from a proliferative state to a neuron-like phenotype upon stimulation with NGF. To visualize the early events of this process, we analyzed the subcellular distribution of the growth-associated protein GAP-43 (neuromodulin), a well-known marker of axonal growth cones and neuronal plasticity [28,29]. GAP-43 immunoreactivity appeared predominantly at the plasma membrane of the characteristic rounded non-differentiated PC12 cells in the absence of NGF (Figure 1a, left). This is consistent with GAP-43 involvement in membrane dynamics and cytoskeletal remodeling [30]. After 24 h of NGF exposure, the cells exhibited a marked morphological transformation, extending slender neurite-like projections from the soma. As expected, GAP-43 redistributed toward the distal ends of these newly formed neurites, delineating regions reminiscent of developing growth cones (Figure 1a, right). The fluorescence intensity of GAP-43 was quantified across different cellular regions revealing that the signal was enriched in early neurites by more than twofold compared to the average fluorescence of the entire cell (Figure 1b). This confirms preferential localization of GAP-43 at these structures. These observations illustrate NGF induces a morphological transition of PC12 cells from a rounded, proliferative phenotype to a polarized, neuron-like state accompanied by reorganization of GAP-43 distribution during the early stages.

### 2.2. NGF Stimulation Enhances GlyT2–GAP-43 Colocalization at Early Neurites

**Figure 2:**
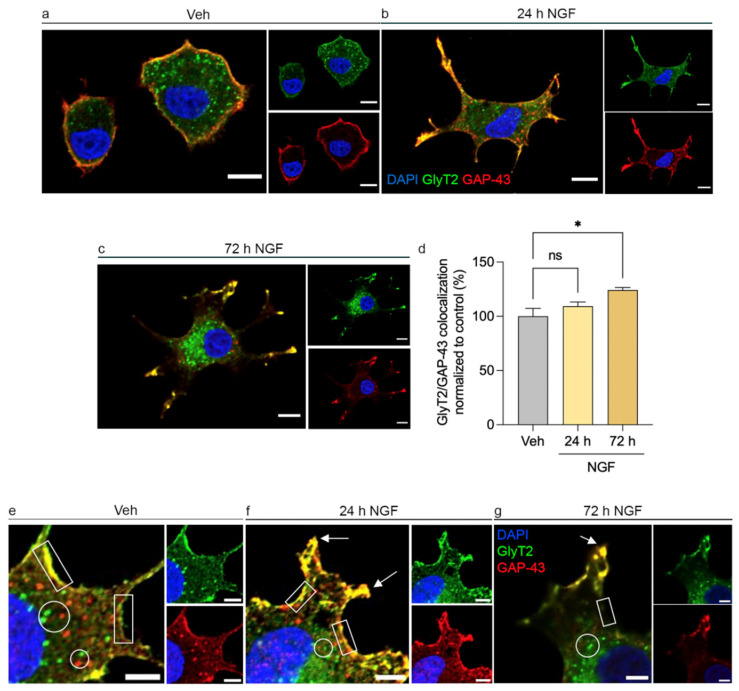
NGF stimulation enhances GlyT2–GAP-43 colocalization specifically at the plasma membrane during PC12 differentiation. (**a**–**c**) Representative confocal images of PC12 cells stably expressing GlyT2, treated with NGF (100 ng/mL) for 0 h (Vehicle, Veh), 24 h, and 72 h. NGF exposure induces progressive redistribution of both proteins toward developing neurites. Scale bars: 10 µm. (**d**) Quantification of the Pearson correlation coefficient between GlyT2 and GAP-43 fluorescence signals. Values are normalized to those of vehicle-treated cells (set to 100%). The colocalization increases over time, reflecting coordinated migration of both proteins to neuritic compartments. * *p* < 0.05 indicates significant difference from control in a one-way ANOVA with Dunnett’s comparison test (65 cells counted in each of *n* = 4 independent cell culture preparations). (**e**–**g**) Representative confocal images of PC12 cells stably expressing GlyT2, treated with NGF (100 ng/mL) for 0 h (Vehicle), 24 h, and 72 h. Squares indicate plasma membrane regions, circles mark intracellular areas, and arrows point to early neurites. GlyT2 and GAP-43 remain colocalized at the plasma membrane throughout differentiation, with increasing accumulation at neuritic extensions as NGF treatment progresses. Scale bars: 5 µm.

To investigate the subcellular dynamics of GlyT2 during neuronal differentiation, we first generated PC12 cell clones stably expressing the transporter, as GlyT2 is not endogenously expressed in this cell line [31]. This stable expression system provides a reliable model to examine how GlyT2 distribution evolves as cells transition from the undifferentiated state to a neuron-like phenotype. In the absence of NGF, both GlyT2 and GAP-43 preferentially localized at the plasma membrane and, to a lesser extent, in perinuclear regions of the rounded cells (Figure 2a). This pattern is consistent with a basal, non-polarized state typical of proliferating cells prior to neuronal commitment.

After 24 h of NGF stimulation GlyT2, as did GAP-43, redistributed from the plasma membrane into the new extended short neuritic processes emanating from the soma during the morphological transformation toward a neuron-like phenotype (Figure 2b). At this stage, both proteins displayed a noticeable degree of colocalization along neuritic shafts and within emerging growth cones. After 72 h of NGF exposure, the morphological and molecular polarization of PC12 cells became more evident and cells developed an intricate network of elongated neurites capped by well-defined growth cones. In these mature extensions, GlyT2 and GAP-43 accumulated prominently at the distal tips, delineating the boundaries of developing growth cones (Figure 2c). This spatiotemporal redistribution correlated with a progressive increase in the overlap of fluorescence signals from both proteins, reflecting a time-dependent process of molecular co-targeting to sites of active neurite outgrowth.

Quantitative analysis of GlyT2–GAP-43 colocalization using Pearson’s correlation coefficient confirmed this trend, revealing a significant increase in signal overlap as differentiation proceeded, reaching 24% increase at 72 h (Figure 2d). Together, these observations indicate that NGF not only drives the morphological differentiation of PC12 cells but also promotes the coordinated recruitment of GlyT2 and GAP-43 to neuritic compartments.

### 2.3. GlyT2 and GAP-43 Occupy Overlapping Membrane-Associated Domains in PC12 Cells

To gain deeper insight into the spatial relationship between GlyT2 and GAP-43 during the early stages of NGF-induced differentiation, we analyzed their subcellular localization using confocal microscopy. In the absence of NGF, both proteins exhibited a primarily peripheral pattern presumably labeling the plasma membrane and adjacent cortical regions, where their fluorescence signals largely overlapped (Figure 2e). Within the cytoplasm, GlyT2 and GAP-43 appeared mostly distinct, suggesting that potential interactions between them are more likely to occur at or near the cell surface rather than in intracellular compartments (Figure 2e). As differentiation advanced, their distribution progressively polarized toward the emerging neurites, particularly at their distal ends, where the overlap between both signals intensified (Figure 2f,g). Altogether, these observations indicate that GlyT2 and GAP-43 occupy partially overlapping membrane-associated domains throughout the differentiation process, with increasing accumulation at the tips of developing neurites.

### 2.4. GlyT2 Expression Does Not Induce Spontaneous Differentiation in the Absence of NGF

**Figure 3:**
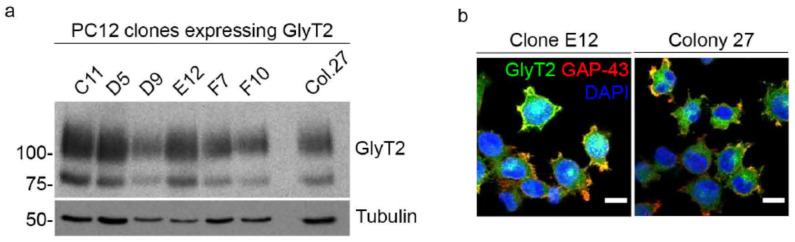
GlyT2 expression in the absence of NGF does not induce spontaneous differentiation in PC12 cells. (**a**) Western blot analysis of several stable PC12 clones expressing different levels of GlyT2. (**b**) Representative immunofluorescence images of two clones shown in (**a**), captured 72 h after plating under basal culture conditions (no NGF treatment). Despite variable GlyT2 expression levels, all clones retained a rounded morphology without neurite formation, indicating that GlyT2 expression by itself is insufficient to trigger spontaneous neuronal differentiation. Scale bars: 10 µm.

Based on the observed localization of GlyT2 in neuritic compartments and its possible association with signaling pathways involved in neuronal development, we next examined whether GlyT2 expression could initiate differentiation in PC12 cells in the absence of NGF. To this end, we generated a panel of stable PC12 clones expressing different levels of GlyT2 (Figure 3a), providing an experimental framework to evaluate whether the magnitude of transporter expression correlates with spontaneous acquisition of neuronal traits under basal conditions.

Immunofluorescence analysis revealed that, irrespective of GlyT2 expression level, all clones maintained the typical rounded morphology of undifferentiated PC12 cells, with no evidence of neurite outgrowth (Figure 3b). Western blot analysis confirmed heterogeneous expression of GlyT2 across the clones, with clone E12 exhibiting nearly threefold higher GlyT2 levels than Colony 27, yet there is no relationship between transporter expression level and morphological differentiation. These results demonstrate that GlyT2 expression *per se* is not sufficient to trigger neuronal differentiation in PC12 cells. This finding underscores the necessity of NGF-mediated signaling to activate the transcriptional and structural programs required for neuronal commitment.

### 2.5. GlyT2 Expression Facilitates NGF-Induced Neuronal Differentiation of PC12 Cells

Having established that GlyT2 expression is insufficient to trigger differentiation in the absence of NGF, we investigated whether GlyT2 could modulate the NGF-induced response. Stable PC12 clones expressing GlyT2, GlyT1, or the dominant-negative GlyT2-S512R HPX point mutant [32] were treated with NGF, and their differentiation was assessed by immunofluorescence and morphological analysis.

**Figure 4:**
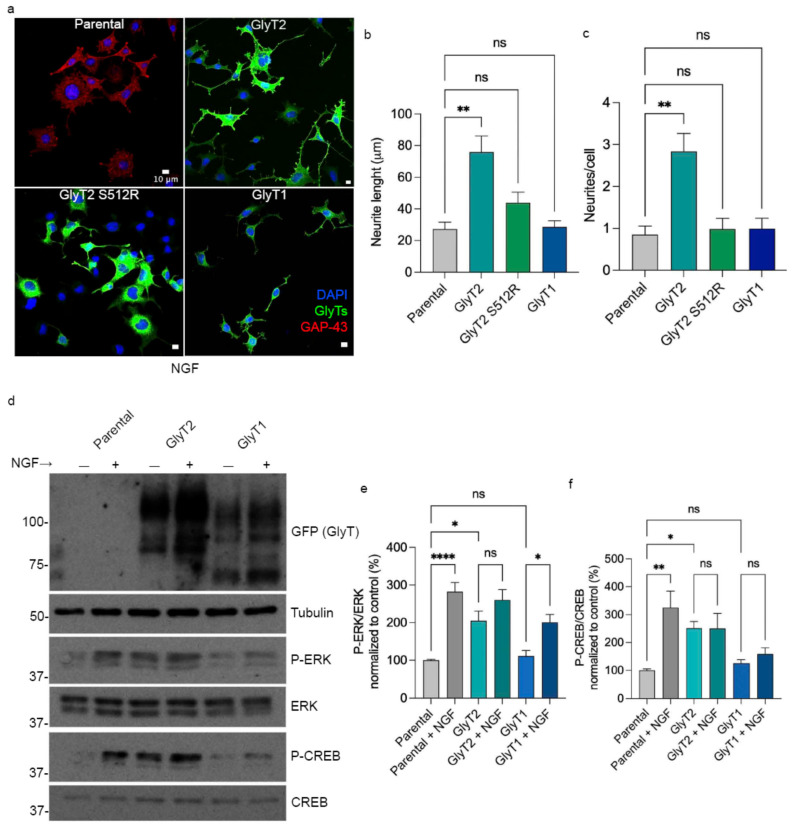
GlyT2 enhances NGF-induced neuronal differentiation of PC12 cells. (**a**) Representative immunofluorescence images of parental PC12 cells and stable clones expressing GlyT2, the dominant-negative mutant GlyT2-S512R and GlyT1, after 72 h of treatment with NGF (100 ng/mL). Scale bars: 10 µm. (**b**,**c**) Quantification of neurite number per cell and average neurite length, respectively, from the images shown in (**a**). ** *p* < 0.01 indicates significant difference from control in a one-way ANOVA with Dunnett’s multiple comparison test (62 cells counted in each of *n* = 3 independent cell culture preparations). (**d**) Western blot analysis of ERK and CREB phosphorylation levels in the same cell lines after 72 h of NGF treatment. (**e**,**f**) Quantification of phosphorylated ERK (P-ERK) and phosphorylated CREB (P-CREB) levels, normalized to vehicle-treated parental cells (set to 100%). GlyT2 expression significantly increases neurite number and length, as well as ERK and CREB phosphorylation, indicating enhanced activation of differentiation-associated signaling pathways. * *p* < 0.05, ** *p* < 0.01, **** *p* < 0.0001 indicate significant difference from control in a one-way ANOVA with Sidak’s multiple comparison test (*n* = 4 independent cell culture preparations).

Upon NGF stimulation, GlyT2-expressing cells increased neurite outgrowth (Figure 4a). The number of neurites per cell increased more than threefold, and neurite length was nearly tripled relative to parental cells and control clones (Figure 4b,c), indicating that GlyT2 expression strongly sensitizes PC12 cells to NGF and promotes a more robust differentiation response. By contrast, cells expressing GlyT1 and GlyT2-S512R mutant-expressing cells exhibited neurite development comparable to parental cells (Figure 4a–c), highlighting that this effect is specific to functional GlyT2. Consistent with these morphological findings, Western blot analysis revealed approximately twofold higher basal phosphorylation levels of ERK and CREB in GlyT2-expressing clones relative to parental controls (Figure 4d). Phosphorylation of ERK and CREB reflects their activation and serves as a hallmark of NGF-driven signaling pathways that promote neuronal differentiation. NGF induces activation of this signaling cascade in all the clones, yet in GlyT2 cells this increase is moderate, although the reached levels are comparable in control and GlyT2-expressing cells. This reinforces the link between GlyT2 expression and the potentiation of neurotrophic signaling (Figure 4d–f) and highlights a facilitating role for GlyT2 in orchestrating neurotrophic responses and promoting neuronal differentiation.

### 2.6. GlyT2 Expression Increases Intracellular Calcium Levels in PC12 Cells

Given the strong association between GlyT2 expression and enhanced neuronal differentiation upon NGF stimulation, we investigated whether the transporter modulates intracellular calcium dynamics, a central regulator of neuronal signaling and morphogenesis [33,34] that has previously been implicated in PC12 cell differentiation [35]. GlyT2 function is known to be calcium-sensitive [36,37], and its colocalization with the growth cone-associated protein GAP-43, a key modulator of calcium homeostasis through its interaction with calmodulin (CaM, [38]), suggested that GlyT2 might influence intracellular calcium balance in differentiating PC12 cells.

**Figure 5:**
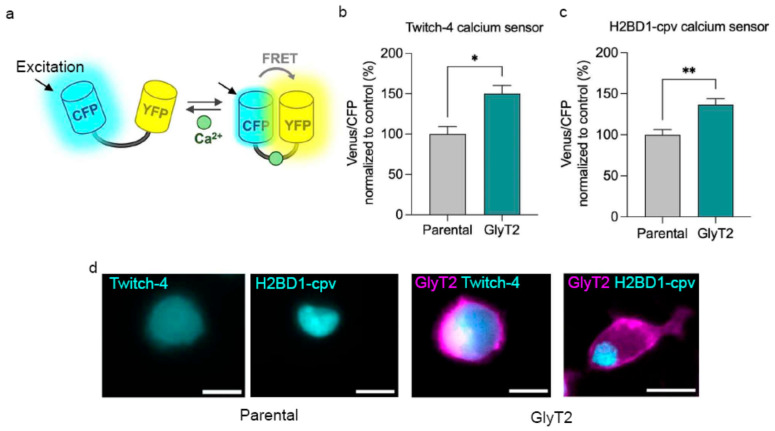
GlyT2 expression elevates basal intracellular calcium levels in PC12 cells. (**a**) Schematic representation of the ratiometric FRET-based calcium sensors used to monitor cytosolic calcium dynamics. (**b**,**c**) Quantification of the Venus/CFP emission ratio (excitation at 430 nm, arrow in (**a**)) in parental PC12 cells and GlyT2-expressing clones transfected with Twitch-4 (**b**) or H2BD1-cpv (**c**) Data are normalized to the mean ratio value of parental cells (set to 100%). GlyT2 expression markedly increases basal cytosolic calcium levels in both sensor systems, indicating enhanced intracellular calcium availability under resting conditions. * *p* < 0.05, ** *p* < 0.01 indicate significant difference from control in a Student’s *t*-test (44 cells counted in each of *n* = 4 independent cell preparations). Scale bars: 10 µm. (**d**) Sucellular localization of GlyT2 and the FRET-based calcium sensors in PC12 cells. H2BD1-cpv is expressed specifically in the nucleus whereas Twitch-4 in the cytoplasm.

To address this question, we employed two independent genetically encoded FRET-based calcium sensors, Twitch-4 [39] and H2BD1-cpv [40], which enable ratiometric measurement of intracellular calcium concentration through changes in energy transfer between donor and acceptor fluorophores (Figure 5a). These probes allow precise quantification of steady-state intracellular calcium differences under basal conditions. Using this approach, we compared parental PC12 cells with stably transfected clones expressing GlyT2.

Both calcium sensors consistently showed that GlyT2-expressing cells exhibit significantly higher basal cytosolic calcium levels than parental controls (Figure 5b,c), with a 50% increase detected using the Twitch-4 sensor and a 37% rise with the H2BD1-cpv probe. Although these probes differ in their subcellular localization (Figure 5d), their localization is independent of transporter expression and both reliably report changes in cytosolic calcium, and each revealed a comparable NGF-induced elevation in intracellular calcium. This suggests that GlyT2 expression intrinsically modifies intracellular calcium homeostasis establishing a higher basal calcium set point that may prime PC12 cells for enhanced activation of calcium-dependent signaling pathways such as ERK and CREB [33,34]. This may facilitate a more efficient neurotrophic differentiation response.

### 2.7. Calmodulin Overexpression Lowers Intracellular Calcium and Attenuates NGF-Induced Differentiation

To further dissect the relationship between intracellular calcium dynamics and neuronal differentiation, we evaluated the effect of CaM overexpression in PC12 cells. In order to reach proper levels of transfection in PC12 cells, we used lentiviral particles containing the pLox plasmid carrying the cDNA of CaM-GFP (material and methods), which allows a stable, long-term modulation of intracellular calcium buffering without the acute toxicity associated with calcium chelators. CaM is a ubiquitous calcium-binding protein that fine-tunes intracellular calcium homeostasis and regulates numerous calcium-dependent signaling cascades critical for neuronal development.

**Figure 6:**
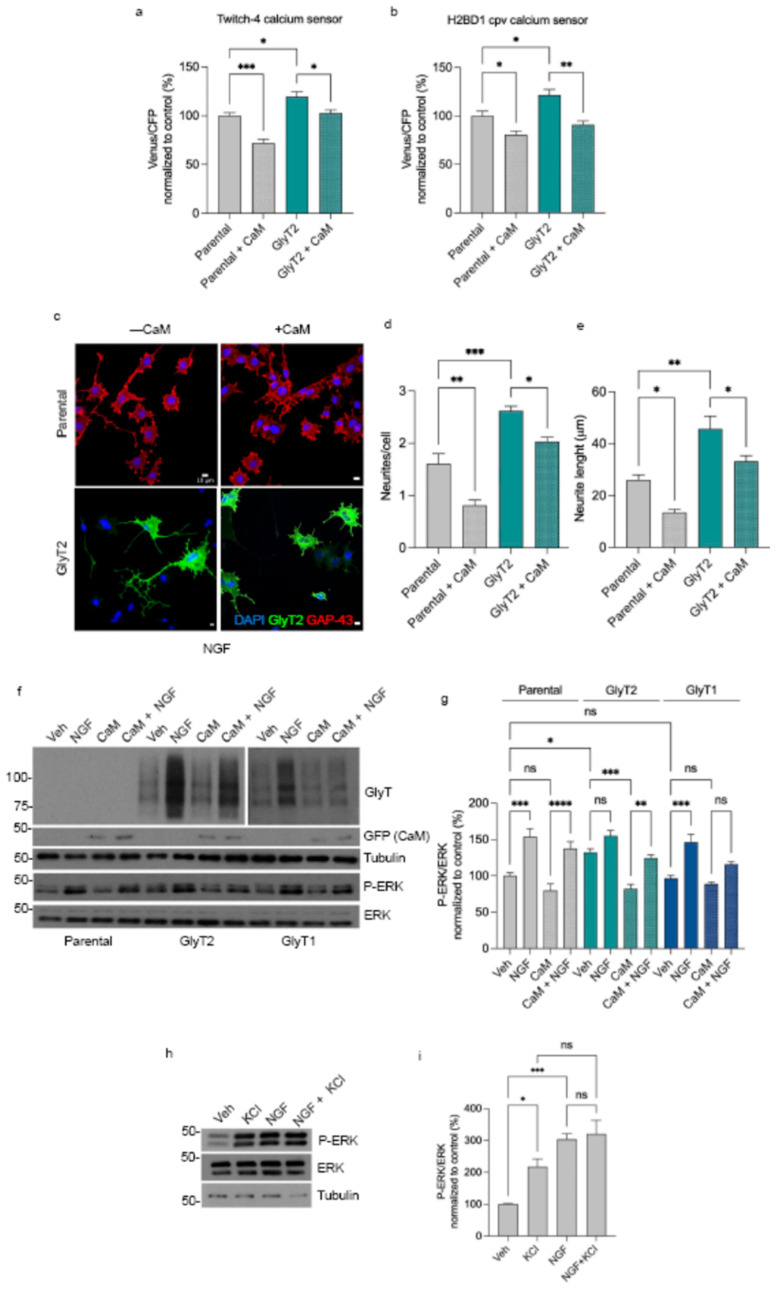
Modulation of intracellular calcium directs NGF-induced neuronal differentiation in PC12 cells. (**a**,**b**) Quantification of cytosolic calcium levels using the ratiometric FRET sensors Twitch-4 (**a**) and H2BD1-cpv (**b**) in parental and GlyT2-expressing PC12 cells. The Venus/CFP emission ratio (excitation at 430 nm) was normalized to the mean ratio value of parental cells (set to 100%) * *p* < 0.05, ** *p* < 0.01, *** *p* < 0.001 indicate significant difference from control in a one-way ANOVA with Sidak’s multiple comparison test (*n* = 4 independent cell culture preparations). Calmodulin (CaM) overexpression significantly reduced basal intracellular calcium levels across both cell types. (**c**) Representative immunofluorescence images of parental and GlyT2-expressing cells with or without CaM overexpression after 72 h of NGF treatment (100 ng/mL). Scale bars: 10 µm. (**d**,**e**) Quantification of neurite number per cell and mean neurite length from the images in (**c**), normalized to control conditions (set to 100%). * *p* < 0.05, ** *p* < 0.01, *** *p* < 0.001 indicate significant difference from control in a one-way ANOVA with Sidak’s multiple comparison test (58 cells counted in each of *n* = 4 independent cell preparations). (**f**) Representative Western blot of phosphorylated ERK (P-ERK) and total ERK in parental, GlyT2-, and GlyT1-expressing PC12 cells treated with NGF for 72 h in the presence or absence of CaM-GFP overexpression. (**g**) Densitometric quantification of P-ERK normalized to total ERK and expressed relative to parental control cells (set to 100%). CaM overexpression lowers cytosolic calcium and attenuates neurite outgrowth and ERK activation, highlighting the requirement of calcium-dependent signaling for efficient PC12 differentiation. * *p* < 0.05, *** *p* < 0.001, **** *p* < 0.0001 indicate significant difference from control in a one-way ANOVA with Sidak’s multiple comparison test (*n* = 3 independent cell preparations). (**h**) Representative Western blot showing P-ERK and total ERK in parental PC12 cells treated for 3 days with vehicle (Veh), KCl (4 mM), NGF, or NGF + KCl, and (**i**) corresponding quantification of P-ERK/ERK levels expressed as percentage of Veh (set to 100%). * *p* < 0.05, *** *p* < 0.001 indicate significant difference from control in a one-way ANOVA with Sidak’s multiple comparison test (*n* = 3 independent cell culture preparations).

In our model, overexpression of CaM markedly reduced basal intracellular calcium levels, as detected by both ratiometric FRET-based sensors Twitch-4 and H2BD1-cpv, in parental and GlyT2-expressing PC12 cells (Figure 6a,b). The concordant reduction observed with both sensors confirms that excessive CaM effectively buffers free cytosolic calcium.

Furthermore, we investigated whether calcium depletion altered the cells’ capacity to differentiate in response to NGF. Immunofluorescence analysis revealed that in CaM overexpressing cells the number of neurites per cell was reduced by approximately 50% (parental PC12 cells) and by 23% (GlyT2-expressing cells), while neurite length decreased by 48% and 27%, respectively (Figure 6c–e). The general reduction of neurite number and neurite length after NGF treatment in reduced calcium conditions exerts a broad inhibition on neuronal differentiation rather than a transporter-specific effect.

At the signaling level, Western blot analysis showed that CaM overexpression led to a decrease in ERK phosphorylation across all the clones (Figure 6f,g). While this reduction did not reach statistical significance in parental or GlyT1-expressing cells, GlyT2-expressing clones displayed a significant 38% decrease in ERK phosphorylation under basal conditions, highlighting the particular sensitivity of GlyT2-mediated differentiation to calcium availability. In addition, to directly test whether calcium elevation is sufficient to enhance ERK signaling in the absence of GlyT2, we chronically depolarized parental PC12 cells with a mild concentration of KCl (4 mM, 3 days) and examined ERK activation by Western blot (Figure 6h,i). Under basal conditions, KCl treatment significantly increased pERK/ERK levels to about 220% of vehicle, whereas NGF stimulation elicited a larger, approximately threefold increase.

Notably, co-treatment with NGF and KCl did not further augment ERK activation beyond NGF alone, suggesting that NGF and depolarization converge on a common ERK pathway whose activation reaches a ceiling under these conditions. These data support a model in which elevated intracellular calcium sustains activation of the ERK pathway and promotes neuronal differentiation, whereas low-calcium conditions dampen calcium-dependent signaling and morphological maturation.

### 2.8. GlyT2 Interacts with GAP-43 and Forms an Indirect Complex with Calmodulin

**Figure 7:**
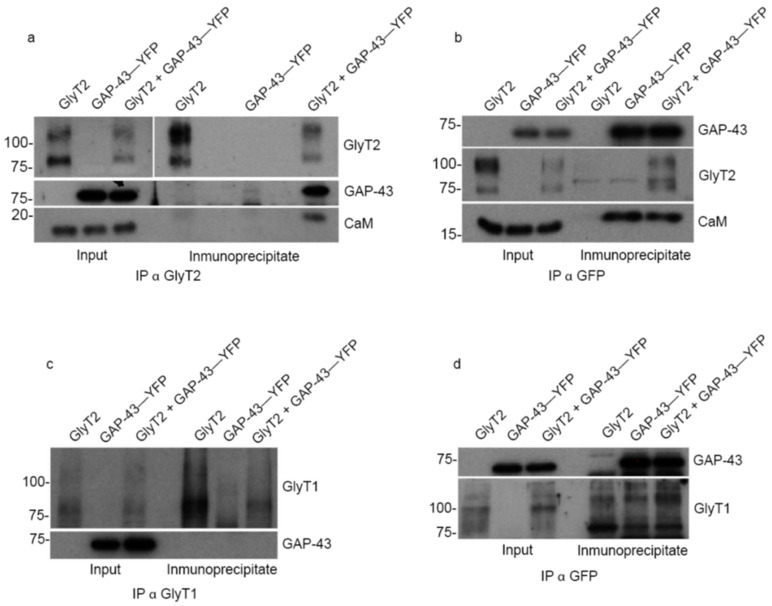
GlyT2, but not GlyT1, interacts with GAP-43 and forms an indirect complex with calmodulin. COS-7 cells were transiently transfected with different combinations of GlyT2 or GlyT1 and GAP-43–YFP to analyze protein–protein interactions by co-immunoprecipitation (co-IP). (**a**,**b**) Reciprocal co-IP assays performed with anti-GlyT2 (**a**) or anti-GFP (GAP-43–YFP) (**b**) antibodies demonstrate a specific and robust interaction between GlyT2 and GAP-43–YFP, as each protein is detected in the pulldown of the other. The use of GAP-43 fused to YFP facilitates its distinction from immunoglobulin heavy chains, ensuring accurate detection of co-precipitated bands. When GlyT2 and GAP-43–YFP were co-expressed, endogenous CaM co-immunoprecipitated with GlyT2 only in the presence of GAP-43, indicating the formation of an indirect GlyT2–GAP-43–CaM complex. (**c**,**d**) Equivalent co-IP assays performed with GlyT1 under identical conditions, using anti-GlyT1 (**c**) or anti-GFP (**d**) antibodies. No detectable interaction between GlyT1 and GAP-43–YFP was observed, confirming the specificity of the GlyT2–GAP-43 association. These results identify GAP-43 as a specific GlyT2-interacting partner and suggest that GAP-43 mediates the recruitment of CaM to form a tri-molecular complex linking GlyT2 to calcium-dependent signaling pathways.

GAP-43 is a pivotal regulator of neuronal growth and synaptic remodeling that modulates intracellular calcium signaling through its ability to bind CaM in its non-phosphorylated form [41]. Given the strong spatial overlap between GlyT2 and GAP-43 observed during PC12 cell differentiation (Figure 2), we explored whether these proteins physically interact, what could potentially explain their coordinated localization and functional convergence on calcium-dependent pathways. To address this question, we took advantage of the COS-7 cell system in which we co-expressed GlyT2 and GAP-43–YFP, and reciprocal co-immunoprecipitation assays were performed. When GlyT2 was immunoprecipitated, GAP-43 was consistently detected in the eluates, and the reverse pulldown yielded the corresponding GlyT2 signal (Figure 7a,b), confirming a robust and specific physical interaction. The use of YFP-tagged GAP-43, which increases its apparent molecular weight, facilitated clear discrimination from immunoglobulin heavy chains and ensured reliable identification of the co-precipitated bands. Moreover, endogenous CaM co-immunoprecipitated with GlyT2 only in the presence of GAP-43 (Figure 7a). In the absence of GAP-43, CaM signal was not detected in GlyT2 pulldowns, indicating that GAP-43 serves as a molecular scaffold bridging GlyT2 to CaM. Thus, GlyT2 does not interact directly with CaM but rather participates in an indirect tri-molecular complex, where GAP-43 couples the transporter to calcium-dependent signaling elements. This configuration may constitute a specialized signaling module that coordinates GlyT2 function with local calcium dynamics and membrane remodeling during neuronal differentiation. To assess the specificity of this interaction, parallel co-immunoprecipitation assays were conducted using GlyT1, a closely related glycine transporter with about 50% sequence identity to GlyT2 but distinct regulatory and expression profiles. Under identical conditions, GlyT1 failed to co-immunoprecipitate with GAP-43, regardless of whether the pulldown targeted GlyT1 or GAP-43 (GFP) (Figure 7c,d). This absence of interaction underscores the molecular specificity of the GlyT2–GAP-43 association and confirms that it is not a general feature of glycine transporters.

Collectively, these results provide biochemical evidence for a specific and functionally relevant interaction between GlyT2 and GAP-43, with GAP-43 mediating the recruitment of CaM to the complex. The formation of this GlyT2–GAP-43–CaM triad defines a potential mechanistic interface linking glycine transport activity to calcium signaling pathways, thereby contributing to the regulation of neuronal differentiation and early neurite development.

### 2.9. GlyT2 Modulates GAP-43 Phosphorylation

Phosphorylation of GAP-43 at serine-41 by protein kinase C (PKC) represents a critical regulatory event that switches GAP-43 from a CaM-binding to a CaM-releasing state, thereby modulating calcium signaling and growth cone dynamics during neuronal differentiation [42]. Given the specific association between GlyT2 and GAP-43 (Figure 7), we examined whether GlyT2 expression influences the phosphorylation status of GAP-43 under basal conditions and following PKC activation.

**Figure 8:**
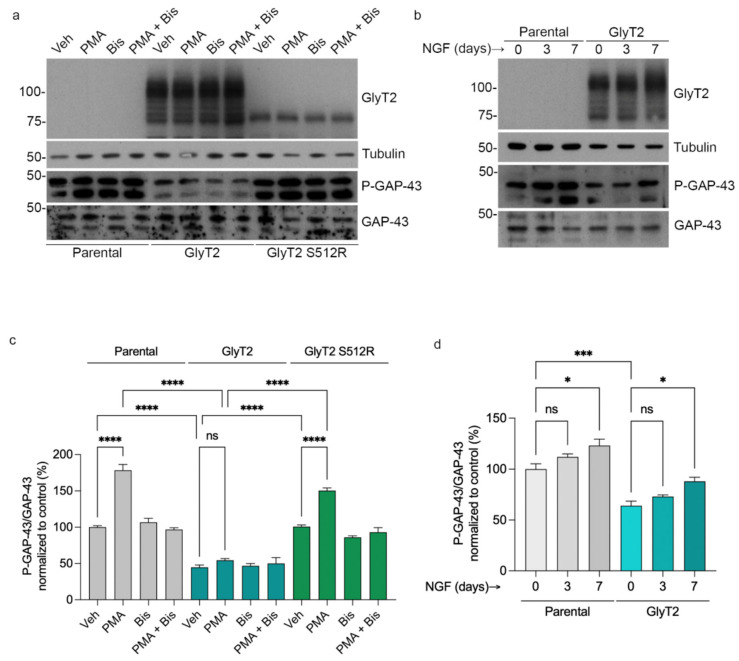
GlyT2 expression reduces GAP-43 phosphorylation and prevents PKC-induced modification. (**a**) Representative Western blots of parental PC12 cells, GlyT2-expressing clones, and the dominant-negative mutant GlyT2-S512R after stimulation with the PKC activator phorbol 12-myristate 13-acetate (PMA, 100 nM, 1 min), preincubation with the PKC inhibitor bisindolylmaleimide I (Bis, 1 µM, 5 min) followed by PMA, or vehicle treatment. In control cells, PMA markedly increases GAP-43 phosphorylation, an effect fully prevented by Bis. In contrast, GlyT2 expression abolishes the PMA-induced phosphorylation response, whereas GlyT2-S512R retains it, indicating that the functional transporter is required for this regulation. (**b**) Western blots of parental and GlyT2-expressing PC12 cells treated with NGF (100 ng/mL) for 0, 3, and 7 days. GAP-43 phosphorylation progressively increases with differentiation in both cell types but remains slightly lower in GlyT2-expressing cells. (**c**,**d**) Densitometric quantification of phosphorylated GAP-43 normalized to total GAP-43 and expressed relative to vehicle-treated parental controls (100%) of the previous Western blots. (**a**,**b**) GlyT2 expression maintains GAP-43 predominantly in a non-phosphorylated state under basal and PKC-stimulated conditions. * *p* < 0.05, *** *p* < 0.001, **** *p* < 0.0001 indicate significant difference from control in a one-way ANOVA with Sidak’s multiple comparison test (*n* = 3 independent cell preparations for each graph).

In control PC12 cells, activation of PKC with phorbol 12-myristate 13-acetate (PMA) induced a robust 78% increase in phosphorylation of endogenous GAP-43, consistent with the established role of PKC in growth cone remodeling ([43], Figure 8a–c). This effect was completely prevented by preincubation with the PKC inhibitor bisindolylmaleimide I, confirming the specificity of the response. Conversely, in PC12 cells stably expressing GlyT2, PMA failed to elicit a comparable increase in GAP-43 phosphorylation, indicating that GlyT2 expression dampens PKC-mediated modification of GAP-43 protein (Figure 8a–c). Furthermore, cells expressing the dominant-negative GlyT2-S512R mutant retained substantial, though attenuated, phosphorylation response of approximately 49%, demonstrating that wild-type GlyT2 is required for this regulatory effect (Figure 8a–c). These observations suggest that GlyT2 maintains GAP-43 predominantly in its non-phosphorylated state, favoring its interaction with CaM, and possibly stabilizing early stages of neurite initiation.

To examine the temporal dynamics of GAP-43 phosphorylation during differentiation, we analyzed the levels of phosphorylated GAP-43 after 0, 3, and 7 days of NGF treatment. In both parental and GlyT2-expressing PC12 cells, GAP-43 phosphorylation gradually increased over time (Figure 8b–d), consistent with its role in later stages of neuronal maturation and synaptic development [44]. Although the overall phosphorylation levels remained lower in GlyT2-expressing cells at every time point, phosphorylation of GAP-43 was increased over time under NGF treatment. These findings indicate that GlyT2 does not prevent the NGF-dependent phosphorylation of GAP-43 required for neuronal maturation, and suggest that GlyT2 finetunes the balance between phosphorylated and non-phosphorylated GAP-43 during differentiation.

### 2.10. GlyT2 Sustains NGF-Induced Actin Cytoskeleton Reorganization During Late Differentiation

**Figure 9:**
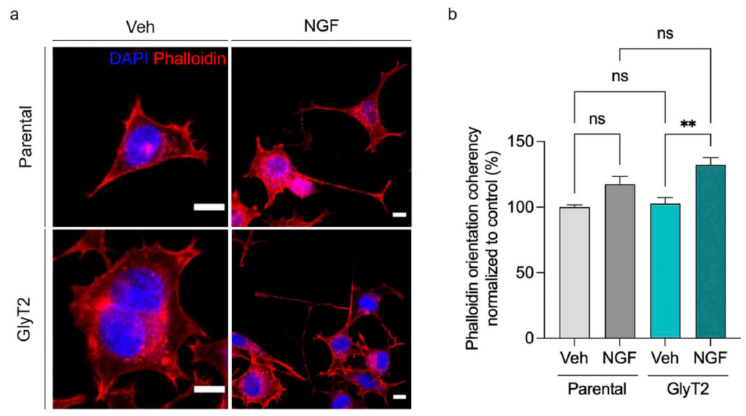
GlyT2 expression sustains actin cytoskeleton reorganization during differentiation. (**a**) Representative immunofluorescence images of parental PC12 cells and GlyT2-expressing clones cultured for 7 days in the presence or absence of NGF (100 ng/mL) and stained with phalloidin to visualize filamentous actin. Scale bars: 10 µm. (**b**) Quantification of phalloidin signal orientation coherency across the four experimental conditions. Data are normalized to vehicle-treated parental controls (set as 100%). Only GlyT2-expressing cells show a significant increase in actin orientation coherency upon NGF treatment. ** *p* < 0.01 indicates significant difference from control in a one-way ANOVA with Sidak’s multiple comparison test (30 cells counted in each of *n* = 4 independent cell preparations).

We investigated whether GlyT2 expression influences actin cytoskeleton remodeling during PC12 differentiation, specifically testing whether it modulates the ability of phosphorylated GAP-43 to reorganize F-actin structures. Parental PC12 cells and GlyT2-expressing clones were treated with vehicle or NGF for 7 days and stained with phalloidin to visualize F-actin (Figure 9a). Under basal conditions, both cell types showed comparable orientation coherency values, indicating similar actin organization in the absence of differentiation cues (Figure 9b). However, following NGF treatment, GlyT2-expressing cells displayed significantly higher coherency than parental cells, reflecting a stronger alignment of actin bundles along a predominant orientation (Figure 9b). This increased anisotropy of F-actin is consistent with a more advanced differentiation state and supports a role for GlyT2 in facilitating cytoskeletal remodeling, likely mediated by phosphorylated GAP-43, associated with neurite stabilization and maturation.

### 2.11. GlyT2 Facilitates Neurite Extension in Primary Cortical Neurons

**Figure 10:**
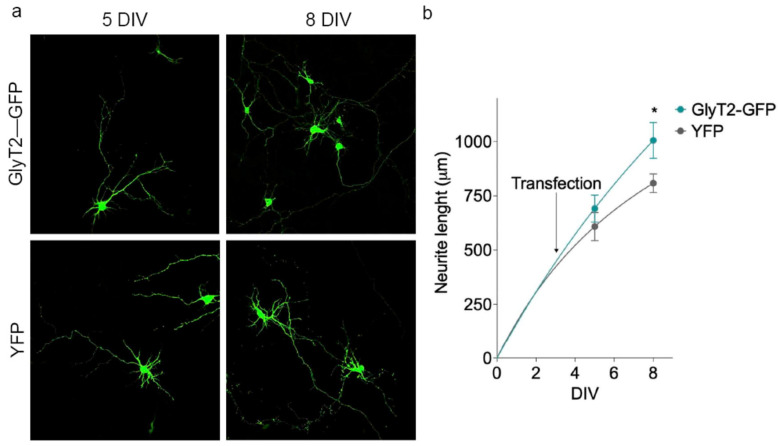
GlyT2 expression facilitates neurite outgrowth in primary cortical neurons. (**a**) Representative immunofluorescence images of embryonic rat cortical neurons transfected at DIV3 with either GlyT2-GFP or YFP alone and fixed at DIV5 and DIV8, showing GFP/YFP fluorescence to visualize transfected neurons. Scale bars: 10 µm. (**b**) Quantification of neurite length in GlyT2-GFP- and YFP-expressing neurons at DIV5 and DIV8. Data represent mean neurite length (µm) ± SEM; 40 neurons were analyzed per experimental condition and time point. * *p* < 0.05 indicates significant difference from control in a Student’s *t*-test (40 neurons were analyzed per experimental condition and time point).

To finally test whether our observations in PC12 cells extend to a more physiological neuronal context, we transiently expressed GlyT2 in cerebral cortex primary neurons, a brain area lacking GlyT2 expression (Figure 10). Neurons were transiently transfected at DIV3 with either GlyT2-GFP or YFP alone and neurite length was quantified at DIV5 and DIV8. Under these conditions, GlyT2-GFP-expressing neurons displayed significantly longer neurites than YFP-expressing control neurons at both time points, indicating that short-term GlyT2 expression is sufficient to facilitate neurite elongation also in primary cortical neurons and thus reinforces the pro-differentiative role of GlyT2 suggested by our PC12 data.

## 3. Discussion

Type III HPX (OMIM 614618) is part of the broader HPX (startle disease) spectrum, caused by mutations in the *SLC6A5* gene encoding GlyT2. Hyperexplexia shows a perinatal window of severity with symptoms usually decreasing after the first year of life. When affecting GlyT2, the disease is more likely associated with developmental delay than when other genes, such as *GLRA1*, are involved [5,6]. This, together with the fact that the expression of GlyT2 precedes the establishment of glycinergic activity in several brain regions [18], and the reported role for glycine as regulator of neurogenesis in different animal models [16,17], suggests that GlyT2 could play a role during early nervous system development.

In this work we have investigated the role of GlyT2 in neurite outgrowth and neuronal differentiation using PC12 cells. For this purpose, we stably expressed the transporter in this system, and examined its behavior upon treatment of the cells with NGF. In these conditions, PC12 cells arrest proliferation, extend neurite-like processes, and adopt morphological and gene expression changes characteristic of neurons [21]. This NGF-induced differentiation paradigm is widely used to study neurogenesis, neuronal survival, and plasticity [20,23]. Our data proved that GlyT2 is clearly implicated in growth cone progression and facilitates neuronal differentiation as the cells stably expressing GlyT2 exhibit increased expression of neuronal differentiation markers and enhanced neurite outgrowth as compared to parental PC12 cells or cells expressing alternative transporters.

One notable feature of GlyT2-expressing cells is their elevated cytosolic calcium levels. This increase may result from the sodium- and calcium-homeostatic activity proposed for GlyT2 through its physical and functional interactions with ion-regulatory proteins, such as the Na^+^-/K^+^-ATPase [45], and the PMCA/NCX [37]. When these GlyT2 cells were induced to lower internal calcium through calmodulin (CaM) overexpression, they also reduced the number and length of neurites and the expression of differentiation markers. This supports that elevated calcium levels are required to activate Ca^2+^/CaM-dependent pathways such as the ERK/CREB axis and, consequently, the transcriptional program driving neurite outgrowth and neuronal differentiation. The reason why CaM overexpression in a high-calcium environment reduces intracellular calcium in our experimental conditions could be due to the fact that excess CaM may exceed the available Ca^2+^, leaving a substantial fraction of CaM molecules partially unsaturated and therefore less capable of forming signaling-competent Ca^2+^/CaM complexes, although capable of functioning as calcium buffers. This reduces free cytosolic calcium, as confirmed by our FRET-based sensors. In addition, we also found that chronically elevating intracellular Ca^2+^ in parental PC12 cells by exposing them to a mild depolarizing concentration of KCl increased basal ERK phosphorylation. This observation indicates that Ca^2+^ elevation in the absence of GlyT2 is sufficient to enhance ERK activation in parental cells, reinforcing the view that GlyT2-dependent calcium changes facilitate, rather than uniquely define, the Ca^2+^-driven differentiation program. In fact, the causal relation between calcium elevation and differentiation is consistent with earlier work in PC12 cells showing that membrane depolarization and Ca^2+^ influx activate ERK/MAPK signaling and promote neurite outgrowth, supporting a permissive role of cytosolic calcium in this differentiation model [46].

Our data additionally identify a novel member of GlyT2 interactome, since we found the transporter interacts physically and functionally with the growth-associated protein, GAP-43. Upon NGF treatment, PC12 cells expressing GlyT2 show a progressive redistribution of both proteins toward the growing neurites, which supports the notion that their coordinated localization contributes to early membrane remodeling and cytoskeletal dynamics underlying neurite formation. Our observations indicate that GlyT2 and GAP-43 colocalize specifically at the plasma membrane throughout the differentiation process, with increasing accumulation at the tips of developing neurites. This is in accordance with the fact that these two proteins are lipid raft-resident [47,48], and the functional interplay between the transporter and the growth-associated protein we uncovered potentially supports the early steps of neuronal morphogenesis. Unlike GAP-43, GlyT2 does not interact directly with CaM but rather participates in an indirect tri-molecular complex, where GAP-43 couples the transporter to calcium-dependent signaling elements. Our finding that GlyT2 modulates GAP-43 phosphorylation dynamics, protecting the protein from PKC-dependent modification under basal conditions, suggests that GlyT2 in the initial stages restrains full differentiation by keeping GAP-43 in a predominantly non-phosphorylated state, thereby limiting its contribution to cytoskeletal remodeling [41]. Treatment of PC12 cells with NGF induces a rise in GAP-43 phosphorylation. This increase is likely driven by NGF-dependent sustained PKC activation overcoming the protective effect of GlyT2 on GAP-43 [49], constituting a key inflection point that helps trigger the differentiation process in GlyT2-expressing cells. It has been shown that GAP-43 phosphorylation, in combination with an increase in calcium levels, reduces affinity for CaM [50,51] and released CaM may participate in calcium-dependent mechanisms and ERK/CREB activation. This may act synergistically with phosphorylated GAP-43 to coordinate actin reorganization, neurite elongation, and growth cone maturation during the terminal phases of PC12 differentiation [52]. We propose that GlyT2 sets a primed state in PC12 cells by elevating basal activation of ERK and CREB, but this signaling alone is not sufficient to drive full differentiation in the absence of GAP-43 phosphorylation. By keeping GAP-43 phosphorylation low, GlyT2 imposes negative feedback that restrains its own pro-differentiative effect until an appropriate NGF stimulus is present. Upon NGF treatment, robust PKC activation overrides this protective brake, allowing GAP-43 phosphorylation to increase, thereby enabling cytoskeletal remodeling, GAP-43 recruitment to neurites, and the execution of the differentiation program. Accordingly, the established role of phosphorylated GAP-43 in actin dynamics is compatible with the enhanced reorganization and alignment of actin bundles observed in GlyT2-expressing cells during late differentiation [28,41,42]. In this model, GlyT2-expressing cells differentiate earlier and more efficiently because they are already in a basally activated state and only require NGF to rapidly trigger the full transcriptional and structural cascade of neuronal differentiation. In this framework, GlyT2 acts as a temporal regulator that limits GAP-43 phosphorylation by maintaining it in a predominantly non-phosphorylated state under basal conditions while simultaneously enhancing NGF sensitivity and downstream pathways, such as ERK and CREB, required for neurogenesis. This hypothesis is also consistent with our observation that GlyT2 expression in the absence of NGF does not initiate differentiation, but it markedly increases the NGF sensitivity of PC12 cells, thereby facilitating neurotrophic responses and promoting neuronal differentiation.

In addition to the PC12 model, we performed initial translational validation in primary embryonic cortical neurons by transiently expressing GlyT2. In this more physiological neuronal context, GlyT2-expressing neurons displayed longer neurites than YFP-expressing controls at early time points, indicating that GlyT2 can also facilitate neurite elongation in primary neurons and supporting the notion that our findings are not restricted to the heterologous PC12 system.

In conclusion, in agreement with the fact that HPX patients achieve successful neurodevelopment, even in the absence of GlyT2 [53], in this study we propose that the transporter seems not to be critical for neurogenesis. This also agrees with gene deletion studies showing mice deficient in GlyT2 are apparently normal at birth and seemingly lack developmental abnormalities [3]. However, here we disclose a new role for GlyT2 as a facilitator of neuronal differentiation, which may provide an etiological basis for the developmental delay observed in a subset of HPX patients [10]. Whether this possibility is indeed actual has to be studied further. In this report we show that the dominant-negative HPX mutant, S512R, that is retained in the endoplasmic reticulum and does not reach the plasma membrane [32], prevents the facilitating effect on neurogenesis exhibited by the wild-type. Our PC12 cells stably expressing the mutant behave as parental cells or cells expressing GlyT1, a GlyT2 homologous transporter we found is devoid of any role in neurodifferentiation. This new GlyT2 feature seems to be due to its proper localization in plasma membrane lipid rafts rather than to its transport function, since cells treated with a GlyT2 transport inhibitor do not seem to alter their differentiation capability. However, this subject will be a matter of further research, as well as the contribution of the GlyT2-GAP-43 interaction in the maintenance of internal calcium concentrations. The novel GlyT2 role must be studied to know to which extent other HPX variants retain or lack this GlyT2 function. This is important for the individual patients harboring these mutations and can guide future research with personalized therapeutic relevance.

## 4. Materials and Methods

### 4.1. Cell Culture

PC12 cells (ATCC CRL-1721, Manassas, VA, USA) kindly provided by Dr. J. Diez-Guerra (CBM, Madrid, Spain) were maintained in Roswell Park Memorial Institute (RPMI) medium supplemented with 5% fetal bovine serum (FBS), 5% horse serum, 2 mM L-glutamine, and 2 mM non-essential amino acids. Cultures were kept at 37 °C in a humidified atmosphere containing 5% CO_2_. Cells were passaged every 72 h, before reaching full confluence, as cell–cell contact leads to detachment and cell death; subculturing was therefore performed at approximately 60% confluence.

COS-7 cells (ATCC CRL-1651, Manassas, VA, USA) and HEK-293T (ATCC-CRL-3216, Manassas, VA, USA) cells were grown in Dulbecco’s modified Eagle’s medium (DMEM) supplemented with 10% FBS, 2 mM L-glutamine, and 2 mM non-essential amino acids, under the same incubation conditions. Cells were subcultured every 72 h when reaching approximately 80% confluence.

### 4.2. Transient Transfection

Transient transfection of COS-7 and PC12 cells was performed using high-molecular weight polyethylenimine hydrochloride (PEI MAX, MW 40,000; Polysciences, Inc., Warrington, PA, USA, Cat# 24765-1). Cells were transfected at 70–80% confluence. For PC12 cells, culture plates were pre-coated with poly-L-lysine (PLL) to enhance adhesion at high density. DNA–PEI complexes were prepared in serum-free medium (DMEM for COS-7 and HEK-293T cells, and RPMI for PC12 cells) at a ratio of 2 µL PEI per µg DNA, following the manufacturer’s recommendations, and incubated for 20 min at room temperature before being added to the cells. After 4 h at 37 °C and 5% CO_2_, the medium was replaced with complete culture medium containing serum. Cells were used for experiments 48 h post-transfection.

### 4.3. Lentiviral Particle Production and Infection

Lentiviral particles were generated in HEK-293T cells to induce heterologous expression of the proteins of interest in PC12 cells. Briefly, HEK-293T cells were co-transfected with the transfer plasmid pLox carrying the cDNA of interest (encoding calmodulin–GFP under the human synapsin promoter), the packaging plasmid, and the envelope plasmid pMD2.G using polyethylenimine (PEI MAX, MW 40,000; Polysciences, Inc., Warrington, PA, USA, Cat# 24765-1; DNA:PEI ratio 1:2). DNA–PEI complexes were prepared in Opti-MEM (Gibco, Thermo Fisher Scientific, Waltham, MA, USA) and incubated for 20 min at room temperature before being added to the cells. After 4 h, the medium was replaced with Neurobasal™ medium (Gibco, Thermo Fisher Scientific, Waltham, MA, USA). Viral supernatants were collected 48 h post-transfection, filtered through a 0.45 µm membrane, aliquoted, and stored at −80 °C until use. For infection, 75 µL of viral aliquot were added directly to each well of a 12-well plate containing PC12 cells.

### 4.4. Generation of Stable PC12 Cell Lines

Stable PC12 cell lines expressing GlyTs were generated from a parental PC12 cell line as described elsewhere [31]. Cells were first transfected with the plasmid pEGFP-C1 (Clontech, Inc., Sa Jose, CA, USA) containing GFP-GlyT2, GFP-GlyT2-S512R or GFP-GlyT1 followed by antibiotic selection with 100 µg/mL G418 sulfate (Gibco, Thermo Fisher Scientific, Waltham, MA, USA) to enrich transporter expressing cells. During several weeks of culture in the presence of G418, GlyTs expression was monitored by immunofluorescence or Western blot. For high-expressing clones obtained from colony #27, they were isolated by limiting dilution, expanded, and screened for GlyT2 levels. The clones exhibiting the highest expression levels selected for subsequent experiments.

### 4.5. PC12 Cell Differentiation

PC12 cells were cultured on plates pre-coated with poly-L-lysine and rat tail collagen. Neuronal differentiation was induced by treatment with 100 ng/mL nerve growth factor (NGF) in DMEM supplemented with 10% horse serum. The medium containing NGF was refreshed every 48 h, and cells were processed at the indicated time points for subsequent experiments.

### 4.6. Pharmacological Treatments

Cells were treated with nerve growth factor (NGF, 100 ng/mL, Sigma-Aldrich, St. Louis, MO, USA, Cat# N6009), the protein kinase C activator phorbol 12-myristate 13-acetate (PMA, 100 nM, Sigma-Aldrich, St. Louis, MO, USA, Cat# P1585), the PKC inhibitor bisindolylmaleimide I (1 µM, Calbiochem, Merck Millipore, Darmstadt, Germany 203290, Cat# 203290) or potassium chloride (KCl, 4 mM, Sigma-Aldrich, St. Louis, MO, USA, Cat# P3911) as indicated in each experiment.

### 4.7. Western Blot Analysis

Cells were collected, protein concentration was determined by Bradford assay (Bio-Rad Laboratories, Hercules, CA, USA) on a 0.2 N NaOH-lysed aliquot, and the remaining material was lysed in RIPA buffer for further analysis. Equal amounts of protein (5–10 µg) were mixed with loading buffer and denatured at 37 °C for 30 min. Proteins were separated by SDS-PAGE (7% or 12.5% acrylamide gels) and transferred to nitrocellulose membranes using a semi-dry system. Membranes were blocked with 5% skim milk in PBS for 1 h at room temperature and incubated overnight at 4 °C with primary antibodies: anti-calmodulin (Abcam, Cambridge, UK, rabbit, 1:5000, Cat# ab45689), anti-CREB (Merck Milipore, Darmstadt, Germany, mouse, 1:5000, Cat# 6D9035), anti-ERK (Santa Cruz Biotechnology, Dallas, TX, USA, 1:5000, rabbit, sc-94), anti-Phospho-CREB (Merck Milipore, Darmstadt, Germany, rabbit, 1:1000, Cat# 06-519), anti-Phospho-ERK (Merck Milipore, Darmstadt, Germany, rabbit, 1:2500, Cat# 05-797R), anti-Phospho-GAP-43 (Merck Milipore, Darmstadt, Germany, rabbit, 1:2000, Cat# 07-430), anti-GAP-43 (home-made, rabbit, 1:3000, generously provided by Dr. Díez-Guerra), anti-GFP (ChromoTek, Planegg, Germany, rabbit, 1:1000, Cat# PABG1), anti-GlyT2 (home-made, rabbit, 1:500), anti-Tubulin (Sigma-Aldrich, St. Louis, MO, USA, mouse, 1:2000, Cat# T6074). HRP-conjugated secondary antibodies—anti-rabbit (Bethyl Laboratories, Montgomery, TX, USA, goat, 1:8000, Cat# A120-401P), anti-mouse (Thermo Fisher Scientific (Pierce), Waltham, MA, USA, rabbit, 1:8000, Cat# 31452)- and Clarity™ Western ECL Substrate (Bio-Rad Laboratories, Hercules, CA, USA) were used for detection. Chemiluminescent signals were captured on film and quantified using Image Lab™ software 6.1 (Bio-Rad Laboratories, Hercules, CA, USA) [9].

### 4.8. Immunofluorescence in Cultured Cells

Cells were seeded on 10 mm coverslips pre-coated with poly-L-lysine and processed as described [9]. After washing with PBS, cells were fixed with 4% paraformaldehyde for 20 min at room temperature and permeabilized/blocked in PBS containing 10% FBS and 0.1% Triton X-100 (Sigma-Aldrich, St. Louis, MO, USA) for 90 min. Primary and secondary antibody incubations were performed in PBS with 1% FBS for 1 h at room temperature: anti-GAP-43 (home-made, rabbit, 1:3000), anti-GFP (Roche, Mannheim, Germany; distributed by Sigma-Aldrich, St. Louis, MO, USA, mouse, 1:100, Cat# 11814460001), anti-GlyT2 (home-made, rat, 1:500), anti-rat Alexa Fluor 488 (Thermo Fisher Scientific, Waltham, MA, USA, donkey, 1:500 Cat# A-21208), anti-mouse Alexa Fluor 488 (Thermo Fisher Scientific, Waltham, MA, USA, donkey, 1:500, Cat# A-21202), anti-rabbit Alexa Fluor 555 (Thermo Fisher Scientific, Waltham, MA, USA, donkey, 1:500, Cat# A-31572), followed by nuclear staining with DAPI (1:5000, 5 min). F-actin was visualized by staining with phalloidin Alexa TRITC (Santa Cruz Biotechnology, Dallas, TX, USA, 1:500, Cat# sc-301530). Coverslips were rinsed, mounted with Mowiol 4-88 (Sigma-Aldrich, St. Louis, MO, USA), and images were acquired using a Nikon A1R+ (Nikon Corporation, Tokyo, Japan) confocal microscope with high-sensitivity GaAsP detectors and six diode laser lines. Image processing and analysis were performed with Fiji(ImageJ) 2.3 (National Institutes of Health, Bethesda, MD, USA). The images were processed with a 2.0-pixel median filter, and the threshold applied was automatically determined by the JACoP plugin (Fiji distribution). Pearson’s value of correlation was obtained with JACoP by comparing the two thresholded channels and measuring the correlation between them. The value can range from −1 to 1, the latter representing maximal correlation and colocalization (two identical images). To assess the organization of the actin cytoskeleton, F-actin bundle alignment was quantified using the OrientationJ plugin in Fiji 2.3 Phalloidin-stained images were converted to grayscale and individual cells were selected as regions of interest (ROIs). OrientationJ computes, for each ROI, the local dominant orientation of actin fibers and a coherency index that reflects how strongly pixel orientations cluster around this predominant angle. High coherency values indicate an anisotropic, well-aligned actin network, whereas low coherency values correspond to a more isotropic, disorganized arrangement of F-actin bundles.

### 4.9. Immunoprecipitation

Protein–protein interactions were analyzed by immunoprecipitation using mild detergent conditions to preserve native complexes [32]. Cells were lysed in PBS containing 0.2% IGEPAL^®^ CA-630 (Sigma-Aldrich, St. Louis, MO, USA, Cat# I3021), 0.1 mM PMSF (Roche, Mannheim, Germany), and a protease inhibitor cocktail (1:250, Sigma-Aldrich, St. Louis, MO, USA, Cat# P7626 and P8465) for 90 min at room temperature with gentle rotation. Lysates were cleared by centrifugation, and equal amounts of protein (determined by Bradford assay, Bio-Rad Laboratories, Hercules, CA, USA) were precleared with protein G–Sepharose beads (NeoBiotech Co., Ltd., Seoul, Republic of Korea, Cat#: NB-45-00037-5) for 30 min at room temperature. Supernatants were then incubated overnight at 4 °C with the appropriate primary antibody—anti-GFP (ChromoTek, Planegg, Germany, rabbit, Cat# PABG1), anti-GlyT1 (home-made, rabbit), anti-GlyT2 (home-made, rabbit)—followed by incubation with protein G–Sepharose beads for 90 min. Immunocomplexes were washed three times with PBS containing 0.2% IGEPAL and eluted in loading buffer at 75 °C for 15 min before analysis by Western blot.

### 4.10. Live-Cell Calcium Imaging in PC12 Cells

PC12 cells were seeded on 20 mm glass-bottom plates (Ibidi, Gräfelfing, Germany) and transfected with ratiometric FRET calcium sensors (Twitch-4 [39] or H2BD1-cpv [40]), with or without co-expression of the protein of interest. Before imaging, cells were incubated in complete Hank’s medium at 37 °C. Fluorescence images were acquired using an inverted fluorescence microscope equipped with a 60× oil immersion objective and an ORCA-Fusion sCMOS camera (LED illumination, Colibri 7; Zeiss Microscopy GmbH, Jena, Germany). FRET ratios (Venus/CFP) were calculated from processed images using Fiji(ImageJ) 2.3 (National Institutes of Health, Bethesda, MD, USA) to quantify intracellular calcium levels.

### 4.11. Seeding, Transfection and Culture of Primary Cortical Neurons

Primary cortical neurons were prepared from embryonic day 18 (E18) rat embryos. Briefly, cortices were dissected in ice-cold HBSS, meninges removed, and tissue was dissociated by incubation with trypsin (0.05%, 16 min, 37 °C) followed by gentle trituration through fire-polished Pasteur pipettes. Cells were plated onto poly-L-lysine–coated glass coverslips (12 mm) in 24-well plates at a density of 100.000 cells/cm^2^ in Neurobasal medium supplemented with 2% B27. Cultures were maintained at 37 °C in a humidified 5% CO_2_ incubator, and half of the medium was replaced every 3–4 days. At 3 days in vitro (DIV3), neurons were transiently transfected with plasmids encoding GlyT2-GFP or YFP alone (control). Transfections were performed using Lipofectamine 2000 (Thermo Fisher Scientific, Waltham, MA, USA) according to the manufacturer’s instructions, with a DNA:Lipofectamine ratio of 1:2.5 in Neurobasal medium without supplements. After 3 h, the transfection mix was replaced with conditioned culture medium. Neurons were allowed to express the constructs until DIV5 or DIV8, as indicated. For immunofluorescence staining, neurons were processed as described above (Section 4.8). Neurite outgrowth was analyzed in neurons expressing either GlyT2-GFP or YFP. For each condition and time point, neurite length was measured in 40 randomly selected transfected neurons. Neurite length per neuron was quantified from single-plane images using Fiji/ImageJ. Images were calibrated using the microscope pixel size, and neurites were traced with the segmented line tool from the soma border to the tip of the neurite; the resulting length was expressed in µm. Only neurons that displayed a healthy morphology were included in the analysis.

### 4.12. Statistical Analysis

Statistical analyses were performed using GraphPad Prism 10 (RRID:SCR_002798). Data are presented as mean ± SEM from at least three independent experiments. Statistical significance between two groups was assessed using unpaired two-tailed Student’s *t*-tests. For multiple group comparisons, one-way or two-way ANOVA was conducted followed by Sidak’s or Dunnett’s multiple comparisons post hoc tests. Normality and homogeneity of variances were verified before performing parametric tests. Differences were considered statistically significant at *p* < 0.05 (*p* < 0.05 *, *p* < 0.01 **, *p* < 0.001 ***, *p* < 0.0001 ****); “ns” denotes non-significant differences. No test for outliers was conducted. Mean values along with the standard error of the mean (SEM) of at least 3 experiments were represented in the graphs.

## Data Availability

The original contributions presented in this study are included in the article. The raw data supporting the conclusions of this article will be made available by the authors upon request. Further inquiries can be directed to the corresponding author.

## Abbreviations

The following abbreviations are used in this manuscript:

ANOVA	Analysis of variance
ATCC	American Type Culture Collection
Bis	Bisindolylmaleimide I
CaM	Calmodulin
CBM	Centro de Biología Molecular
CFP	Cyan Fluorescent Protein
CNS	Central Nervous System
CREB	cAMP Response Element-Binding protein
DAPI	4′,6-diamidino-2-phenylindole
DMEM	Dulbecco’s modified Eagle’s medium
ERK	Extracellular signal-Regulated Kinase
FBS	Fetal Bovine Serum
FRET	Fluorescence Resonance Energy Transfer
GABA	Gamma-Aminobutyric Acid
GAP-43	Growth-Associated Protein 43
GFP	Green Fluorescent Protein
*GLRA1*	Glycine Receptor, Alpha 1
GlyR	Glycine Receptor
GlyT	Glycine Transporter
HPX	Hyperekplexia
HRP	Horseradish Peroxidase
MCIN	Ministerio de Ciencia e Innovación
NCX	Sodium Calcium Exchanger
NGF	Nerve Growth Factor
OMIM	Online Mendelian Inheritance in Man
PBS	Phosphate-Buffered Saline
PEI	Polyethylenimide
PKC	Protein Kinase C
PLL	Poly-L-Lysine
PMA	Phorbol 12-Myristate 13-Acetate
PMCA	Plasma Membrane Calcium ATPase
PMSF	Phenylmethylsulfonyl Fluoride
ROI	Region of Interest
RPMI	Roswell Park Memorial Institute
*SLC6A5*	Solute Carrier Family 6, Member 5
